# A Turn-On–Type Fluorescence Resonance Energy Transfer Eco-friendly Method for Nitazoxanide Quantification in Pharmaceutical Dosage Form and Spiked Plasma: Evaluation of Greenness Profile Using Different Assessment Tools

**DOI:** 10.1007/s10895-022-03072-4

**Published:** 2022-12-21

**Authors:** Eman A. Mostafa, Ehab F. Elkady, Mai A. El-Didamoony, Hany A. Batakoushy

**Affiliations:** 1 Pharmaceutical Chemistry Department, Faculty of Pharmacy, Cairo University, Kasr El-Aini St, Cairo, 11562 Egypt; 2 Pharmacist at Ministry of Health, Zagazig, Egypt; 3 Department of Pharmaceutical Analytical Chemistry, Faculty of Pharmacy, Menoufia University, Shebin Elkom, 32511 Egypt

**Keywords:** Nitazoxanide, Reduction, Spectrofluorimetry, Dosage form, Plasma, Green profile

## Abstract

A brand-new class of anti-infective drugs that work against bacteria, viruses, and protozoan parasites is nitazoxanide and related thiazolides. Thiazolides have also been shown to cause cell cycle arrest and apoptotic cell death in cancer cells in recent years. In this study, an eco-friendly, spectrofluorimetric technique that is verified, easy, and sensitive has been proposed for quantifying nitazoxanide (NTZ), a broad-spectrum antiparasitic drug. When NTZ is reduced with zinc (Zn) powder in an acidic media, a highly fluorescent product is produced. To get the highest sensitivity, different experimental conditions impacting the response were examined and optimized. Following excitation at 299 nm, scanning of the fluorescent product was done at 440 nm. The intensity of the fluorescence was proportional to the drug concentration in the range of 0.1–0.6 μg/mL. The approach was validated according to International Conference on Harmonization (ICH) guidelines, and the outcome was satisfactory. The detection and quantitation limits were calculated to be 0.013 and 0.038 μg/mL, respectively. The suggested technique was successful in analyzing commercially available NTZ dosage forms. Furthermore, the proposed technique was used to assess NTZ levels in human plasma and it was bio-analytically validated according to European Medicines Agency (EMA) guidelines. The suggested method can be used in quality control laboratories as well as in pharmacokinetic studies. In order to picture the green profile of the developed method, four greenness assessment tools have been applied. National Environmental Methods Index (NEMI), analytical Eco-Scale Assessment (ESA), Green Analytical Procedure Index (GAPI) and Analytical Greenness metric (AGREE) are the relatively most widely used metrics. So, they were utilized to perform a detailed greenness comparison between the proposed method and some of the reported methods for the determination of NTZ. The developed method was found to be an excellent green method with the highest AGREE score.

## Introduction

**Fig. 1:**
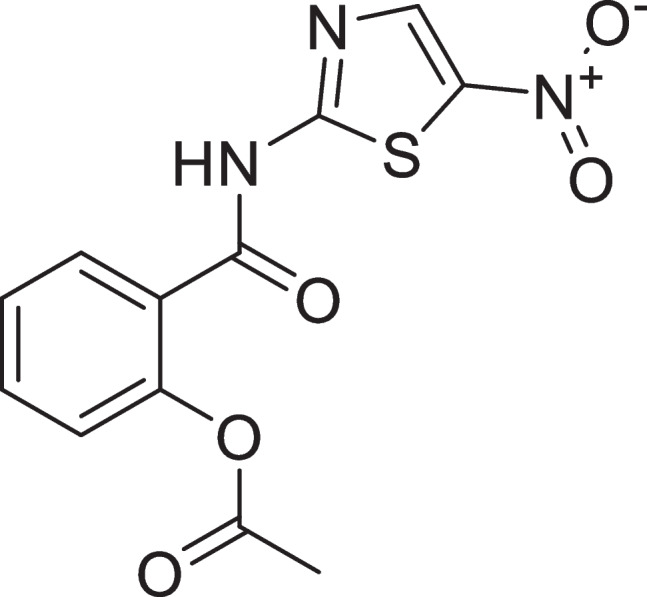
Chemical structure of nitazoxanide (NTZ)

A unique class of broad-spectrum anti-parasitic drugs for the treatment of gastrointestinal infections includes; Nitazoxanide (NTZ), (2-acetolyloxy*-N*-(5-nitro-2-thiazolyl) benzamide), (Fig. 1) and structurally related thiazolides has been recently developed [1]. With a remarkable safety profile, NTZ, the parent drug of the thiazolides family, has been utilized to treat patients afflicted with a range of parasite protozoa and helminth [2]. Additionally, in-vitro investigations have demonstrated its efficacy in treating microbial infections caused by both aerobic and anaerobic bacteria. [1, 2]. The drug must be monitored for quality assurance in preparations and to achieve the best therapeutic doses while limiting the risk of toxicity [3]. There are few analytical literature reviews for estimation of NTZ such as spectrophotometry [4–6], HPLC [7–9], HPTLC [4, 10, 11], and stripping voltammetry [12–15]. Also, some bioanalytical methods for quantitation of NTZ were reported [16–18]. These current analytical methods for NTZ determination require expensive instruments or much time for sample pre-treatment. Also, the reported electrochemical methods always need electrode pre-modification. Thus, we found it urgent to establish a simple and time-saving approach for NTZ determination [19–24]. Fluorimetry is a sensitive and relatively selective approach that has been applied for NTZ analysis. NTZ does not have a native fluorescence, when the stated medication was reduced with Zn powder in an acidic media, a highly fluorescent product was produced. This was the basis for the analytical procedure that was proposed [25–27]. The developed method was successfully applied to NTZ in its commercial dosage form and human plasma. The laboratories performing quality control can use this study. In addition, it could be used in pharmacokinetic studies and therapeutic drug monitoring.

In the present time, one of the primary goals of analytical laboratories is to pursue the movement towards green analytical chemistry (GAC). The twelve basic rules of GAC are the principles on which all greenness assessment tools depend on [28–30]. GAC focuses mainly on achieving the balance between reducing the environmental hazards of analytical methods and restoring the high quality of its results. The environmental hazards may be toxic solvents and/or reagents, energy-efficient instruments, huge amounts of toxic waste or danger to human health and the environment [31, 32]. Several assessment tools have been developed for the evaluation of influences of analytical processes on the environment [33]. The most widely used metrices are National Environmental Methods Index (NEMI) [34], analytical Eco-Scale Assessment (ESA) [35], Green Analytical Procedure Index (GAPI) [36] and Analytical Greenness metric (AGREE) [37]. In order to get a deeper view and detailed green profile of the compared analytical procedure, it is recommended to combine the four methods upon assessing and/or comparing the greenness of analytical method (s) [38–44]. So, the aim of the current study is to develop an eco-friendly analytical procedure which achieve the balance between being sensitive from one side and being eco-friendly from the other side.

## Experimental

### Instruments

Fluorescence spectra were acquired by an FS5 spectrofluorimeter (Edinburgh, UK) with a 150 W xenon lamp source for excitation. Also, with 1-cm quartz cell and connected to Fluoracle^®^ software. The slit widths were set to 2 nm and scanning speed 1000 nm/min. Analytical digital balance (Switzerland) has been used. pH measurements were done with an Adwa (model AD1030) pH meter.

### Materials and Reagents

All the reagents used were of analytical grade. NTZ (Purity 99.8%) was provided from National Organization for Drug Control and Research, (NODCAR), Giza, Egypt. Nanazoxid^®^ tablets, each tablet contains 500 mg NTZ produced by Future pharmaceutical industries, Cairo, Egypt was purchased from local pharmacy. Zinc metal dust powder was obtained from BDH Chemicals (Poole, UK). Other solvents used in this method were from Sigma-Aldrich in the United States and were of analytical-grade A. These included methanol, ethanol, acetonitrile, deionized water, isopropyl alcohol, and butanol. El Nasr Chemical, Co., (Egypt) provided the sodium hydrogen phosphate, hydrochloric acid, and citric acid. Borate and acetate buffers, the pH range of (6.0–10.0), and (3.0–5.0) respectively, were prepared at a concentration of 0.2 M. Britton Robinson Buffer (0.04 M) in pH range of (2.0–7.5) was prepared according to USP guidelines [45]. Human plasma samples collected in heparinized tubes and were kindly provided from the blood bank (Menoufia University Hospitals, Menoufia, Egypt) and stored at -20ºC nominal until analysis before being thawed.

### Standard Solution Preparation

Standard stock solution of NTZ was prepared by dissolving a quantity of NTZ powder equivalent to 100 mg in 5 mL methanol and completed to the mark with distilled water in 100 mL volumetric flask to produce a standard stock solution of 1000 µg/mL. Further dilutions were done with ultra-purified water to give a final concentration in the range of 0.1–0.6 μg/mL.

### General Procedures

#### Construction of Calibration Curve

An aliquot of 1.0 mL of NTZ working solution was transferred into a 100 mL conical flask, along with 1.5 mL of conc. HCl and 0.4 gm of Zn powder. This mixture was allowed to stand for 15 min while being gently shaken. The product was then filtered, and precise volumes of filtrate (reduced NTZ standard solutions) from 0.1 to 0.6 µg/mL were transferred into a series of 10.0 mL volumetric flasks after completed to the mark by methanol. Following excitation at 299 nm, the fluorescence intensity was measured at 440 nm. To obtain the calibration curve, fluorescence intensity was plotted against NTZ concentrations in µg/mL. The corresponding regression equation was then calculated.

#### Assay of Nitazoxanide in Tablets

After being ground, the contents of ten Nanazoxid^®^ pills were weighed and well combined. In a 25 mL volumetric flask, a weighed portion of the powder corresponding to 25 mg of the cited drug was transferred, and 5 ml of methanol was then added. Different volumes of the filtrate were quantitatively transferred into a 10.0 mL volumetric flask after the flask contents had been sonicated for 15 min and completed with methanol. Then the procedure utilized to construct calibration curve mentioned in "Construction of Calibration Curve" was followed.

#### Bioanalysis of Nitazoxanide in Human Plasma

Into 10 mL centrifuge tube, serial working solutions were prepared then an accurate volume of 100 μL NTZ standard solutions was quantitatively added to 400 μL of blank plasma, then 2.0 ml acetonitrile were added as a protein precipitation, and vortex mixed for two minutes followed by centrifugation at 5000 rpm for 20 min. Different volumes of the supernatant were used at a general analytical procedure "Construction of Calibration Curve".

### Method Validation

According to ICH guideline Q2 (R1), the optimized spectrofluorimetric technique for NTZ determination in dosage form was validated by assessing linearity, accuracy, precision, specificity, LOD (limit of detection) and LOQ (limit of quantitation) [46]. Additionally, the suggested method for determining NTZ in plasma underwent bio-analytical validation in accordance with EMA guidelines [47].

## Results and Discussion

**Fig. 2:**
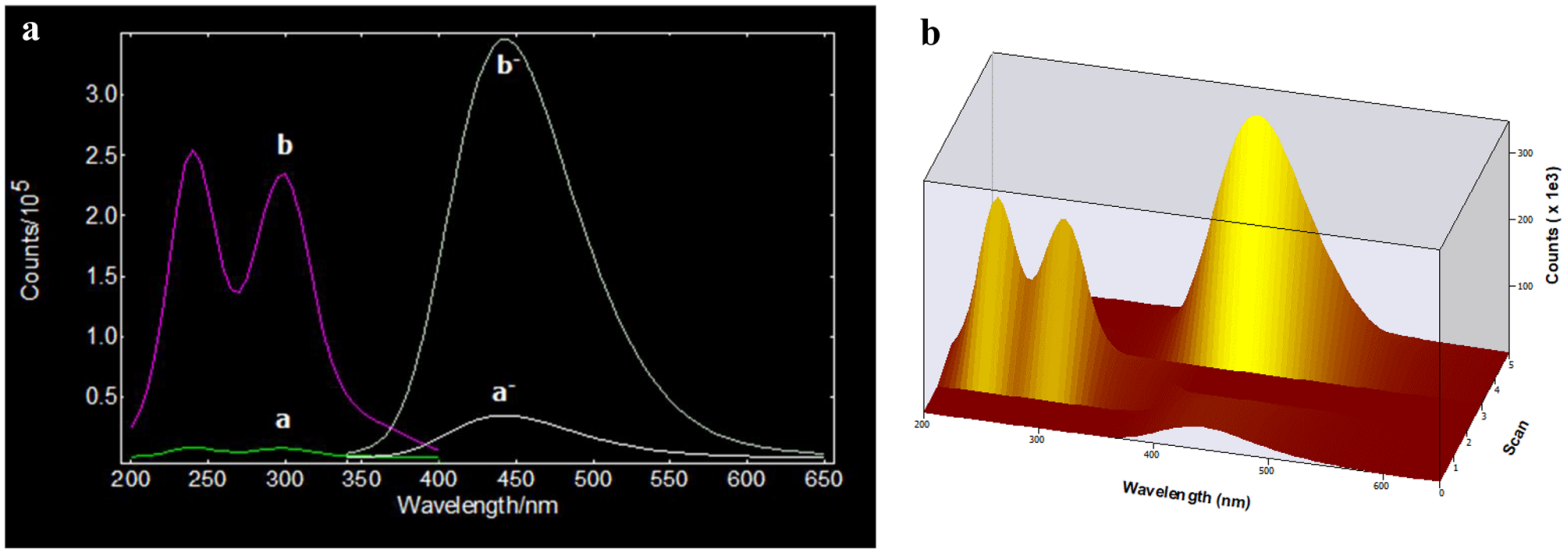
**A** Excitation (**a**, **a-**) and emission (**b**, **b-**) spectra of blank and RNTZ product, respectively. **B** Three-dimension plot for Excitation and emission spectra of blank and RNTZ product, respectively

**Scheme 1:**
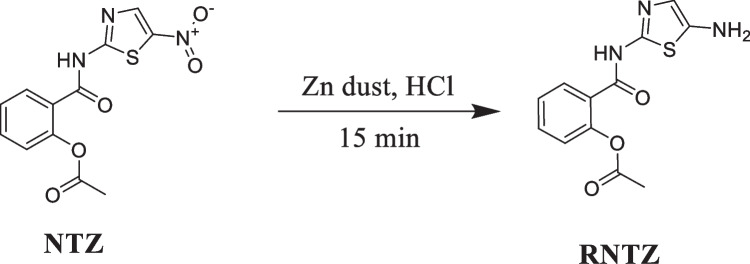
Reduction of NTZ to fluorescent product, RNTZ

Scanning of NTZ's fluorescence spectrum revealed that despite containing a nitro group, it did not produce any fluorescence. The proposed mechanism relies on reducing the NTZ nitro group with Zn in presence of hydrochloric acid (HCl) to the equivalent amino group, Scheme 1. It was noted that, NTZ after reduction process has high fluorescence intensity as presented in (Fig. 2).

### Variable Optimization

The influence of several experimental variables on fluorescence intensity was investigated and optimized. The quantity of Zn powder, volume of concentrated HCl, and reduction duration are the parameters used to carry out the reduction process. To select the most appropriate reduction system providing the highest fluorescence intensity, these factors were examined and optimized.

#### Zinc Powder Amount

**Fig. 3:**
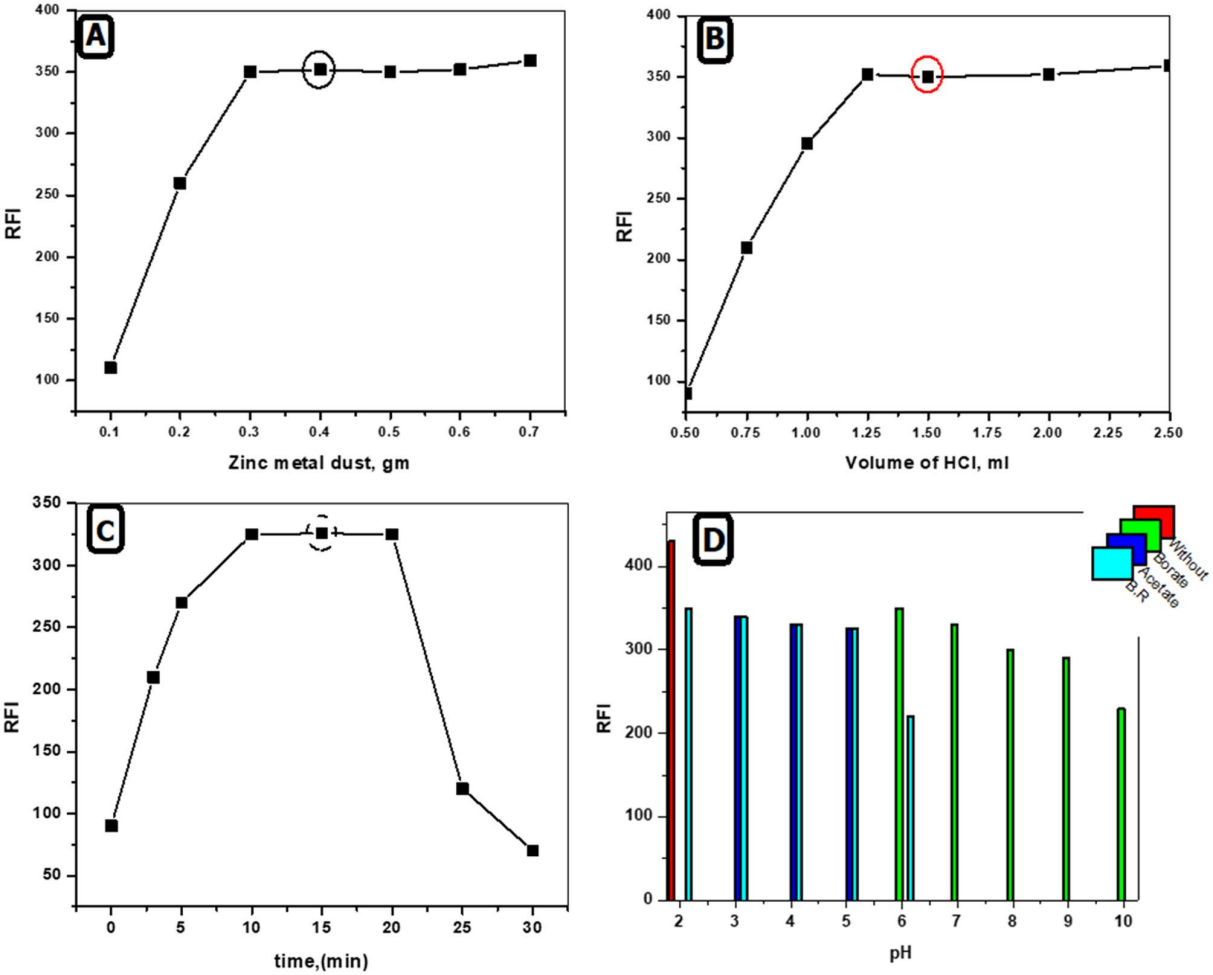
**A** Effect of zinc metal dust amount on the formation of RNTZ fluorophore (0.3 μg/mL). **B** Effect of volume of HCl on the formation of RNTZ fluorophore (0.3 μg/mL). **C** Effect of reaction time on the formation of RNTZ fluorophore (0.3 μg/mL). **D** Effect of various buffer on the formation of RNTZ fluorophore (0.3 μg/mL)

The influence of the amount of Zn powder (0.1–0.6 gm) was studied. It was observed that increasing the amount of Zn metal powder resulted in an increase in fluorescence intensity till 0.3 gm then no more increase was obtained (Fig. 3A), therefore 0.4 gm was used through the proposed analytical method.

#### Hydrochloric Acid Volume

The influence of volume of Conc. HCl, (0.5- 2.5 mL) on the fluorescence intensity was observed. As the volume of HCl increased, the fluorescence intensity increased up to 1.25 mL after this value no more increase was obtained. It was noted that, 1.5 mL of HCl is the optimum volume for complete reduction of NTZ (Fig. 3B).

#### Reduction Time

The influence of time on the fluorescent product development was observed in the range of (0–30 min) at room temperature. It was noted that the reduction process of RNTZ started immediately, and the reaction product reached maximum fluorescence intensity after 10 min (Fig. 3C). Therefore, the optimum time was chosen at 15 min and the obtained fluorophore remained stable for at least one hour.

#### Effect of pH

Different types of buffers, such as, 0.2 M of both borate, acetate buffers in pH range of 2–10 and 0.04 M Britton–Robinson buffers were examined to study the influence of pH on the fluorescence intensity of RNTZ. The proposed study proved that the best fluorescence intensity was achieved without the need for any of the previously mentioned buffers, as demonstrated in (Fig. 3D). Consequently, no buffer was used during the experiment that produced the highest fluorescence intensity.

#### Effect of Diluting Solvent

**Fig. 4:**
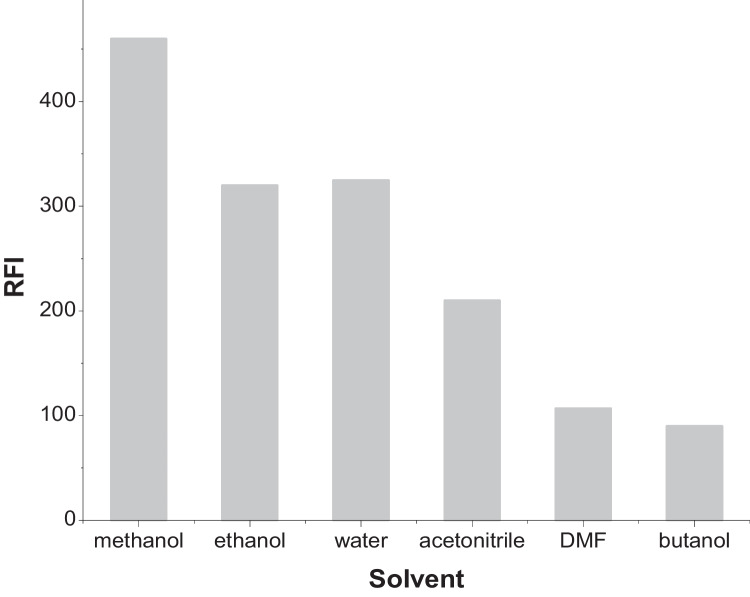
Effect of diluting solvents on the formation of RNTZ fluorophore (0.3 μg/mL)

Several diluting solvents were examined to find out their effect on fluorescence intensity of RNTZ. These solvents were methanol, ethanol, water, acetonitrile, isopropyl alcohol, and butanol. It was noted that methanol was the most suitable solvent to be used, as it had the highest fluorescence intensity with reproducible results as showed in (Fig. 4).

### Methods Validation

The proposed method was validated using ICH for application in pharmaceutical preparation and EMA guidelines for determination in plasma [46, 47].

#### Validation for Pharmaceutical Application

##### Linearity and Range

The general analytical procedure was used to examine a series of standard solutions containing various concentrations of NTZ. The calibration curve was created by plotting the obtained fluorescence intensity against the corresponding drug concentration. The obtained data were subjected to a linear regression analysis, and statistical parameters were calculated in Table 1. The drug concentration was found to be linear with fluorescence intensity in the range of 0.1–0.6 μg/mL, with high linearity (r^2^ = 0.9996).

**Table 1:** Statistical data and quantitative parameters for NTZ determination by spectrofluorimetry for the proposed method

**Parameter**	**NTZ**
λex (nm)	299
λem (nm)	440
Linearity Range (µg/mL)	0.1–0.6
Intercept (a)	271.53
SD of Intercept (Sa)	0.978
Slope (b)	253.71
SD of Slope (Sb)	2.512
Correlation coefficient (r)	0.9998
Coefficient of determination (r^2^)	0.9996
SD of residual (Sy/x)	1.051
Limit of detection (µg/mL)	0.013
Limit of quantitation (µg/mL)	0.038

*SD* standard deviation

##### (LOD) and (LOQ)

LOD and LOQ was used to assess the method's sensitivity. The calculations were done using the following equations: LOD = 3.3 σ / S, LOQ = 10 σ / S where S is the slope of the calibration curve, and σ is the standard deviation of the intercept. The estimated LOD and LOQ were 0.013 and 0.038, respectively. These limits demonstrate that the developed method had high sensitivity as presented in Table 1..

##### Accuracy 

Five concentrations of NTZ within the range of (0.1–0.6 μg/mL) were employed to assess the accuracy of this approach, each concentration being tested three times. The results are provided as a percent recovery ± RSD, as seen in Table 2. The average percent recovery was 99.49 ± 1.69, indicated an acceptable level of accuracy.

**Table 2:** Evaluation of the accuracy of the proposed spectrofluorimetric method of NTZ in pure form

**Sample number**	**Added Conc (µg/mL)**	**Found **^**a**^** Conc (µg/mL)**	**% Recovery ± SD**
1	0.15	0.150	100.64 ± 1.06
2	0.20	0.198	99.05 ± 0.43
3	0.25	0.243	97.49 ± 0.44
4	0.30	0.294	98.16 ± 0.83
5	0.40	0.408	102.12 ± 1.02

*SD* standard deviation

^**a**^ Mean of three replicate measurements

##### Robustness

The robustness of this study was tested by applying minor changes in some experimental parameters (amount of Zn powder, volume of HCl, and reduction time) during the general analytical procedure. These modifications didn’t significantly affect the response, percent recovery indicated that the proposed method is robust. The obtained results were presented in Table 3.

**Table 3:** Robustness of NTZ determination of the proposed method

**Variables**	**Value**	**% Recovery ± SD **^**a**^
**Optimum 100.23 ± 0.44**		
Amount of Zinc powder (0.4 g)	0.35 g 0.45 g	100.64 ± 1.02 99.79 ± 0.83
HCl volume (1.5 mL)	1.25 mL 1.75 mL	99.75 ± 1.2 100.15 ± 0.45
Reduction time (15 min)	10 min 20 min	100.12 ± 0.24 99.85 ± 0.82

^a^ the average of three determinations

##### Specificity

The analytical method's specificity refers to its capacity to judge the analyte response in the presence of any potential contaminants or excipients present in the dosage form. Tablet NTZ analysis revealed no excipient influence. The good recovery % and low standard deviations (SD) proved the high specificity of the proposed method, Table 4.

**Table 4:** Application of the proposed spectrofluorimetric method for assay of NTZ in tablet dosage form

**Parameter**		**Nanazoxid**^**®**^ **500 mg tablets**
	**Proposed method**	**Reported method **[6]
% Recovery	99.68	99.16
Standard deviation (SD)	0.83	0.50
Number of determinations	6	6
t-Value^**a**^	1.34	
f-Value^**a**^	2.75	

^**a**^ tabulated value at 95% confidence limit; t = 2.306 and F = 6.338

##### Statistical Analysis

After development and full validation of the proposed analytical method, it was successfully used for NTZ analysis in its commercial tablets. The results of the proposed method were statistically compared to a reported method [6]. The estimated values of both parameters did not exceed the theoretical values using student's t-test and F-test at 95% confidence level as shown in Table 4.

#### Bioanalytical Method Validation

In addition, bio-analytical validation of the proposed method was performed according to EMA recommendations [47].

##### Selectivity

Six blank plasma samples from different human subjects were analyzed to examine the interference from endogenous matrix. No interference was observed at LLOQ level of the cited drug.

##### Linearity and Lower Limit of Quantitation

Six concentrations of 0.1, 0.2, 0.3, 0.4, 0.5 and 0.6 μg/mL were studied as stated in Experimental "Bioanalysis of Nitazoxanide in Human Plasma". For each concentration, four independent measurements were made. The percentage recovery of NTZ in spiked plasma samples ranged between 98.11 and 101.3%, with RSD values ranging between 0.32 and 1.65%. The results were presented in Table 5.

**Table 5:** Application of the proposed spectrofluorimetric method for assay of NTZ in spiked human plasma

**Parameter**	**Added Conc** **(µg/mL)**	**Found **^**a**^** Conc** **(µg/mL)**	**% Recovery** ± **SD**
Spiked human plasma	0.1	0.09	98.11 ± 1.49
	0.2	0.20	101.3 ± 0.32
	0.3	0.29	99.3 ± 1.65
	0.4	0.39	99.1 ± 0.51
	0.5	0.49	98.6 ± 1.44
	0.6	0.59	98.3 ± 0.45

*SD* standard deviation

^a^ Mean of six determinations

##### Accuracy and Precision

Six-replicate analyses of drug mixture at concentrations of LLOQ, low, medium, and high QC samples (0.10, 0.25, 0.45 and 0.5 μg/mL), respectively in human plasma on the same day were used to assess intra-day accuracy and precision (repeatability). The between-run accuracy and precision of the mixture were determined by analyzing LLOQ, low, medium, and high QC samples on three consecutive days. The method's precision was expressed as a percentage coefficient of variation (CV%). A calibration curve and six replicates of LLOQ, low, medium, and high QC samples were included in each run. According to the results in Table 6, the proposed method has a high accuracy and precision in human plasma.

**Table 6:** Intra-day and inter-day precision results of NTZ in human plasma

**NTZ Conc. (μg/mL )**	Intra-day assay (**n = 6)		Inter-day assay (**n = 18)	
	**Accuracy (%)**	CV*(%)	**Accuracy (%)**	CV*(%)
LLOQ (0.10)	100.24	0.43	99.79	0.62
LQC (0.25)	98.75	1.02	99.01	0.47
MQC (0.45)	98.23	0.82	98.09	0.95
HQC (0.50)	99.30	1.10	99.87	0.93

^*^ CV = coefficient of variation; ^**^ n = number of determinations; ^***^ LLLOQ (lower limit of quantitation), LQC (low QC), MQC (medium QC) and HQC (High QC)

##### Dilution Integrity Test

Sample dilution shouldn't have an impact on the method's accuracy and precision. To show how dilution affects the suggested method, we used 20-fold dilution factor. Human plasma was spiked with (50 μg/mL) of NTZ to produce a concentration of 10 μg/mL (approximate Cmax of NTZ), and then this sample was diluted twenty times with a blank plasma sample. Following 20-fold dilution of the (10 μg/mL) sample, the concentration of NTZ in blood plasma was measured, as well as the accuracy and precision of the results were good. The 20-fold dilution test's coefficient of variation was found to be 1.84%, and the accuracy results were found to be 89.55%.

##### Stability Study

Three aliquots of low and high QC samples were kept at room temperature (25–30 °C) for 6.0 h to determine the short-term stability of plasma samples. The samples were processed, evaluated, and compared to freshly prepared comparison samples with nominal concentrations at the conclusion of the 6.0 h. Three aliquots of each of the low and high QC samples were kept in a deep freezer for 30 days at (-70 ± 5 °C) to test the long-term stability of plasma samples. The samples were processed, analyzed, and contrasted with nominal concentrations at the end of the 30 days.

Three aliquots of each low and high QC sample were processed before being stored at (25–30 °C) for 6.0 h were used to determine the post-preparative stability of plasma samples. Samples were examined and nominal concentrations were compared after 6 h.

Three freeze and thaw cycles were used to determine the impact of freeze and thaw cycles on the stability of plasma samples. Each low- and high-unprocessed QC sample was divided into three aliquots, which were then frozen and thawed three times at (-70 ± 5 °C). Samples are frozen for at least 12 h before each cycle, after which they are allowed to thaw naturally. After the third cycle was finished, the samples were processed, the data was analyzed, and the outcomes were compared to freshly prepared comparison samples. In terms of short-term, long-term, post-preparative, and freeze–thaw stabilities, NTZ was discovered to be stable in human plasma. Table 7 displays the coefficient of variation and accuracy. After bio-analytical validation of the developed method was ascertained, the proposed method's excellent sensitivity allowed the effective in vitro determination of NTZ in spiked plasma.

**Table 7:** Stability of NTZ in human plasma under different conditions

**Stability term**	**NTZ Conc. (μg/mL)**	**Accuracy (%)**	CV* (%)	n**
**Short term**	0.25	100.60	1.16	3
	0.50	99.87	1.09	3
**Long term**	0.25	96.36	1.34	3
	0.50	97.14	0.98	3
**Post-preparative**	0.25	95.66	1.88	3
	0.50	100.35	1.73	3
**Freeze and thaw**	0.25	96.25	1.12	3
	0.50	95.42	1.53	3

^*^ CV = coefficient of variation; ^**^ n = number of determinations

### Green Profile Evaluation

Because they apply to the majority of analytical techniques, NEMI, ESA, GAPI, and AGREE are the comparatively most popular metrics. Those four tools have been used in the current study to estimate the green profile of the developed method. Also, a simple comparison has been performed between the proposed method and some of the previously reported methods for determination of NTZ.

#### National Environmental Methods Index (NEMI)

To begin with, NEMI is the first method selection and parameter comparison tool created by US government agencies. Although it is straightforward and users can quickly grasp the procedure, the information gathered is highly generic, it takes time to complete the NEMI symbol, and it cannot be characterized as semi-quantitative. By simple observation of the pictograms obtained for the developed method and the reported methods, there is almost no difference between them. Only one reported method [10] has 2 green quarters only as it uses a hazardous reagent, Table 8.

**Table 8:** ESA, NEMI, GAPI and AGREE tools for greenness assessment of recently published methods and the developed method for determination of NTZ

**Methods**	**ESA**			**NEMI**	**GAPI**	**AGREE**
**The developed method** **(Dosage form)**	**Reagents**		**Penalty Points**			
		Methanol	6			
		water	0			
		Hydrochloric acid (1.5 ml)	4			
	**Σ**		10			
	**Spectrofluorimetry**		0			
	**Waste**		1			
	**Occupational hazards**		0			
	**Σ**		1			
	**Total Penalty Points**		11			
	**ESA score**		**89**			
**The developed method** **(Spiked plasma)**	**Reagents**	Methanol	6			
		water	0			
		Hydrochloric acid (1.5 ml)	4			
		Acetonitrile	4			
	**Σ**		14			
	**Spectrofluorimetry**		0			
	**Waste**		1			
	**Occupational hazards**		0			
	**Σ**		1			
	**Total Penalty Points**		15			
	**ESA score**		**85**			
[6]	**Reagents**	Acetonitrile	8			
		Water	0			
		Phosphoric acid	2			
	**Σ**		10			
	**UV spectrophotometry**		0			
	**Waste**		1			
	**Occupational hazards**		0			
	**Σ**		1			
	**Total Penalty Points**		11			
	**ESA score**		**89**			
[11]	**Reagents**	Acetonitrile	8			
		Ethyl acetate	4			
		Iso-octane	8			
		Phthalate buffer	0			
	**Σ**		20			
	**HPTLC**		0			
	**Waste**		3			
	**Occupational hazards**		0			
	**Σ**		3			
	**Total Penalty Points**		23			
	**ESA score**		**77**			
[10]	**Reagents**	Acetonitrile	8			
		Methanol	6			
		Ethyl acetate	4			
		Toluene	6			
	**Σ**		24			
	**HPTLC**		0			
	**Waste**		3			
	**Occupational hazards**		0			
	**Σ**		3			
	**Total Penalty Points**		27			
	**ESA score**		**73**			
[8]	**Reagents**	Methanol	12			
		Acetonitrile	8			
		Citric acid	1			
	**Σ**		21			
	**HPLC/UPLC**		1			
	**Waste**		3			
	**Occupational hazards**		0			
	**Σ**		4			
	**Total Penalty Points**		25			
	**ESA score**		**75**			

#### Eco-scale Assessment (ESA)

Secondly, ESA which is the most applicable tool and the one developed mainly for quantification of the green parameters of a method [38]. This tool takes in consideration much more details about the analytical procedures than NEMI does. It depends on its calculations on penalty points which assigned for the method based on the nature of reagents and solvents used, occupational hazards, energy consumed during the process, amount of waste. The result of ESA is a number obtained by subtraction of the total penalty points assigned for the method from100. As the score is closer to 100, then the analytical method is greener where a score of 100 represents an ideal green analytical method. By evaluating the developed method by ESA, it has got a great score of 89 upon applied on dosage form and a score of 85 if applied on spiked plasma. This score defines the proposed method as an excellent green method which was the main target of the current study. Upon applying ESA tool, one could compare the developed method with the reported ones. Only reported method [6] has got the same ESA score of the proposed one but by deep investigation of its details using AGREE as will be discussed later, the developed method has proved to be more eco-friendly method. All ESA scores of the developed method and the reported ones have been described in detail in Table 8.

#### Green Analytical Procedure Index (GAPI)

Thirdly, five pentagrams make up the symbol on which GAPI is based. Each step in the analytical method is expressed by a pentagram to represent its impact on the environment. The severity of the environmental impact is represented by three colors; green, yellow, and red. GAPI has the privilege of combining the advantage of NEMI and ESA together because it gives a rapid summary as well as a detailed analysis of how green certain parts of the analytical process are [32]. The first pentagram which consists of four fields is related to sampling and the developed method and the reported ones are colored in the same pattern. The second pentagram is related to the type of method and is composed of only one field. It is colored red only for the developed method when applied in spiked plasma as it needs sample extraction step. On the other hand, the developed method applied on dosage form and the reported ones are all colored yellow as they all need simple sample treatment steps for sample preparation. This pentagram has got a circle in the middle for all methods as they are quantitative methods. The third pentagram has three fields include extraction scale, consumed reagents, and extra treatments. Concerning the third pentagram, only the developed method got extra red segment because it involves a derivatization step. The fourth pentagram is concerned with the quantity of solvents/reagents utilized and the risks to one's health and safety. The developed method was the only method that consumed less than 10 ml of solvents which results in coloring the field concerned with the volume consumed of the organic solvents with green color. The fifth pentagram deals with the amount of energy used by the tool, potential workplace dangers, waste production, and waste disposal. The best methods in the fifth pentagram were the developed method and the reported method [6] where all segments are colored green in this pentagram, Table 8.

#### Analytical Greenness Metric (AGREE)

The most recent developed greenness assessment tool is AGREE which is introduced in 2020 [37]. The main advantage of AGREE is the simplification of strong and weak sections among the twelve principles of GAC. AGREE depends on the 12 principles of GAC and it is composed of 12 segments. On a scale of 0 to 1, with 1 being the greenest and 0 (red color) the least, each section is colored in accordance with how green it is. The developed method has got the highest score of **0.62** if applied in spiked plasma while it has got a score of **0.59** upon application on dosage form. On the other hand, the reported method [8] has got the least score (0.48) because of the use of large volume of organic solvent and it utilized the most energy-consumed instrument among the compared methods. The obtained pictograms of AGREE scoring of the developed method and the reported methods are presented in Table 8. After investigating the green profile of the developed method, it could be concluded that it is an eco-friendly method which has been defined by ESA as an excellent green method with ESA score of **89**. In addition, it has got the highest AGREE score of **0.62** among the compared methods.

## Conclusion

An eco-friendly, excellent green, simple, sensitive, time saving and cost-effective validated spectrofluorimetric method has been proposed. This study was developed for the quantification of NTZ in its pharmaceuticals and human plasma. Additionally, unlike chromatographic approaches, the new method does not require complicated prerequisites. In addition to its reproducibility, the proposed method could be used in quality control laboratories as well as in pharmacokinetic studies for analysis of the studied drug. The green profile of the developed spectrofluorimetric method was evaluated and it has achieved the highest AGREE score of 0.62 when compared with some of the reported methods.

## Data Availability

The datasets generated during and/or analyzed during the current study are available from the corresponding author on reasonable request.
